# Crusted scabies in a rabbit model: a severe skin disease or more?

**DOI:** 10.1186/s13071-023-05995-8

**Published:** 2023-11-14

**Authors:** Mahmoud Shafeik Sharaf, Ahmad Ali Othman, Amira Elsayed Abdel-Ghaffar, Dareen Mohamed Ali, Mohamed Mahmoud Eid

**Affiliations:** 1 Parasitology Department, Faculty of Medicine, Tanta University, Tanta, Egypt; 2 Pathology Department, Faculty of Medicine, Tanta University, Tanta, Egypt

**Keywords:** Scabies, *Sarcoptesscabiei*, Ivermectin, Fluralaner, Systemic changes

## Abstract

**Background:**

Around 200–300 million people are estimated to be affected by scabies annually worldwide. However, the mechanisms by which this disease may affect the general condition of the host are not entirely clear. The aim of the present study was to evaluate the systemic changes that may accompany crusted scabies in both treated and non-treated experimental animals.

**Methods:**

Male New Zealand rabbits were infected with *Sarcoptes scabiei* var. *cuniculi* and divided into the following three groups: control, ivermectin-treated, and fluralaner-treated. Several methods were used to evaluate the systemic changes, including histopathological examination of the liver, kidney, heart, and spleen, as well as the measurement of serum biochemical parameters and immunological parameters.

**Results:**

Several definite structural and functional changes at the systemic level were revealed, as evidenced by the observed histopathological changes in the tissue sections of internal organs and the highly significant increases in markers of systemic inflammation, serum procalcitonin, and oxidative stress markers. Abnormalities in the liver and renal function results, as well as in the serum lipid profile, were also noted. Additionally, a disorganized immune response was noted, evidenced by a mixed type 1 and type 2 helper T cell response. Although there was notable clinical and parasitological cure in the ivermectin-treated group, the histopathological, biochemical, and immunological markers indicated incomplete resolution. In contrast, the fluralaner-treated group exhibited a nearly complete resolution of changes in these parameters.

**Conclusions:**

We conclude that crusted scabies is a systemic syndrome that can affect several organs besides the skin. Inflammation, oxidative stress, and possibly bacterial infections, are all implicated as underlying mechanisms of tissue damage due to the disease. We recommend that fluralaner, a promising scabicidal agent, should be studied for possible human use, and especially for control programs.

**Graphical Abstract:**

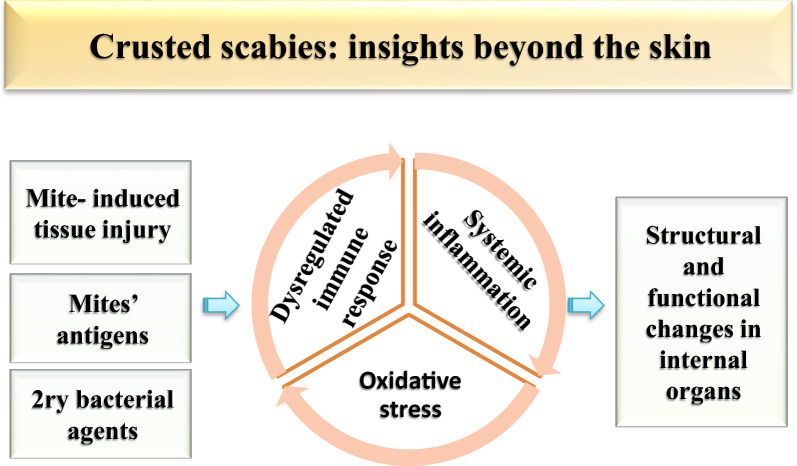

**Supplementary Information:**

The online version contains supplementary material available at 10.1186/s13071-023-05995-8.

## Background

Scabies was recently included by the World Health Organization [1] in its 2021–2030 roadmap for neglected tropical diseases. While there are no published incidence rates for crusted scabies (CS), around 200–300 million people are estimated to be affected by scabies annually worldwide, which is an unacceptably high prevalence for a neglected disease [2]. Even though scabies affects people of all ages globally, children and the elderly in low-resource areas are the most vulnerable, with the highest prevalences, morbidities, and secondary complications [3]. CS usually affects immunocompromised individuals (e.g., those with acquired immunodeficiency syndrome, leukemia, or who are malnourished) [4]. Despite being a less common form of scabies, it is a serious, debilitating form of the disease, which is largely neglected [5].

It is well established that mite antigens that diffuse into the dermis can trigger cellular and humoral immune responses and disturb the balance between type 1 helper T cell (Th1) and Th2 immune responses [6]. Unfortunately, little is known about the systemic changes that occur in the affected host. It is thought that heavy infestation could lead to serious physical deterioration and eventually death of the affected host. The mechanisms by which this disease may affect the general condition of the host are not entirely clear [7].

Ivermectin (IVM) is the only available orally administered drug that is currently approved for treating scabies in humans. It is a macrocyclic lactone that is mostly used in mass drug administration for outbreaks in endemic areas to treat severe crusted forms or poorly compliant patients [8]. Concerns regarding its efficacy and safety in certain situations (e.g., in the treatment of young children, during pregnancy or breastfeeding) have prompted research efforts to discover new alternatives for the treatment of scabies [9–12]. Fluralaner (FLR), a new acaricide of the novel isoxazoline class, has recently gained attention as a promising acaricide against *Sarcoptes scabiei* [13].

To gain a better understanding of the host–parasite interactions in CS at the systemic level, a number of parameters were evaluated in both non-treated and treated experimental animals, with special emphasis on changes in the histopathology of different organs, biochemical parameters, oxidant/antioxidant balance, and immunological parameters. Understanding changes in these parameters may allow the establishment of complementary indicators for the diagnosis and treatment of scabies, especially in resource-poor countries, which could be used to decrease the incidence of erroneous diagnosis and treatment failure.

## Methods

### Animals

Forty 2-month-old parasite-free male New Zealand rabbits, weighing between 1500 and 2000 g, were used in this study. On arrival at the laboratory, the rabbits were randomly allocated separate wire cages in a well-ventilated room at 25 ± 2 °C under a 12/12-h light/dark cycle, and had free access to water and standard food (a commercial pellet diet). The rabbits were allowed to acclimatize to their new surroundings, feed, light/dark cycles, cage mates, and the personnel for 1 week before the induction of infection. 

### Parasite

Based on the rabbit/rabbit model of Casais et al. [14], three naturally infected rabbits suffering from CS were used as a source of *S. scabiei* var. *cuniculi* mites for the experimental induction of CS. Skin was scraped from the edges of lesions with a scalpel till capillary bleeding was evident (the resulting wounds were disinfected with cotton swabs soaked in iodine). The collected scales were placed in petri dishes and incubated at 30 °C for 30 min to enhance the migration of the mites to the surface of the dishes [15]. Then, the dishes were examined microscopically to identify the characteristic morphological features of scabies mites. Mite-infested skin crusts (containing approximately 600–800 mites) were transferred to the ear canals of mite-free rabbits for the induction of infection [12].

### Drugs

IVM was administered as 6 mg Iverzine oral tablets (UNIPHARMA, Egypt) at a weekly oral dose of 0.4 mg/kg body weight for 4 weeks, starting at 8 weeks after the induction of infection [16]. For dose preparation, two tablets were crushed and dissolved in 30 ml distilled water to produce a solution with a concentration of 0.4 mg IVM/ml. Each rabbit received approximately 2 ml of the resulting solution by sucking it from a plastic syringe.

FLR was administered as 250 mg Bravecto chewable tablets (MSD Animal Health, Austria) as a single oral dose of 25 mg/kg body weight, starting at 8 weeks after the induction of infection [17]. For dose preparation, three tablets were crushed and dissolved in 30 ml distilled water to produce a solution with a concentration of 25 mg FLR/ml. Each rabbit received approximately 2 ml of the resulting solution by sucking it from a plastic syringe.

### Experimental design

There were two main experimental phases: the post-infection phase, and the post-treatment phase. The first phase involved the progression of the scabies infestation to the crusted form, while the second phase involved regression of the disease after the induction of treatment. Each phase lasted for 8 weeks. The rabbits were divided into four groups, each of which comprised 10 animals: group I (negative control—not infected, not treated); group II (positive control—infected, not treated); group III (infected, treated with IVM); and group IV (infected, treated with FLR).

Each treated group was isolated from the other groups during the treatment period. Furthermore, the rabbits within each treated group were kept in separate cages but in the same area. Additionally, the surrounding environment was treated twice weekly with deltamethrin (Butox; Intervet) to avoid cross-infestation between treated rabbits [18]. The assessment of different parameters was done at the end of the 8th week post-infection for the positive control group and at the end of the 8th week post-treatment for the IVM- and the FLR-treated groups. Following their clinical and parasitological evaluation, the rabbits were anesthetized by ether inhalation and cardiac puncture was performed for the collection of blood samples (10 ml from each rabbit) in serum-separating Vacutainer tubes. Finally, the rabbits were euthanized by cervical decapitation, skinned, and dissected for the collection of different tissue samples.

### Clinical evaluation

The rabbits were clinically evaluated by observing changes in their body weight and clinical signs of infestation (e.g., pruritus, crusts, and alopecia).

### Parasitological evaluation

Skin scrapes (2 cm^2^) were collected from skin lesions on the pinnae of each rabbit by using a blunt scalpel. Following 30 min incubation at 30 °C, the scrapes were examined microscopically to count the viable mites.

### Histopathological and histochemical evaluation

Tissue samples were carefully collected from the liver, kidneys, spleen, and heart of each rabbit, and preserved in 10% formol saline for histopathological evaluation. The slides were evaluated microscopically, with the pathologist blinded with respect to which group the animal had been allocated. Congo red stain was used for the assessment of amyloid deposition [19], while Masson’s trichrome stain was used for the assessment of fibrosis [20].

### Immunohistochemical evaluation

Immunofluorescence staining of the paraffin-embedded renal sections was done according to Zaqout et al. [21] to assess immune complex deposition. The sections were stained with mouse anti-rabbit immunoglobulin G (heavy and light chains) labeled with Axa Fluor 488 dye (catalogue no. 31584; Invitrogen; Thermo Fisher Scientific, USA) at a dilution of 1:500. Untreated slides with serum were used as negative control slides. The evaluation of immune complexes was done microscopically using a fluorescent microscope (LX-400 Binocular LED Fluorescence Compound Microscope; Labomed, USA), and the Atlas 16MP Cmos USB Camera with PixelPro 3.0 software (Labomed).

### Biochemical and immunological evaluation

Blood samples were allowed to clot within serum-separating Vacutainer tubes for 1 h at room temperature, then centrifuged for 20 min at 3000 r.p.m. Finally, the serum samples were collected in Eppendorf tubes and stored at – 20 °C till use.

The biochemical parameters of evaluation included markers of systemic inflammation and tissue damage [serum levels of serum amyloid A (SAA), C-reactive protein (CRP), lactate dehydrogenase (LDH), and creatine kinase myocardial band (CK-MB)]; serum procalcitonin (PCT); markers of redox status [serum levels of total nitric oxide (NO), malondialdehyde (MDA), reduced glutathione (GSH), and total antioxidant capacity (TAC)]; liver and renal function tests [serum levels of albumin, alanine aminotransferase (ALT), aspartate aminotransferase (AST), alkaline phosphatase (ALP), blood urea nitrogen (BUN), and creatinine (Cr)]; and serum lipid profile [serum levels of total cholesterol (TC), high-density lipoprotein (HDL), low-density lipoprotein (LDL), and triglycerides (TG)]. Additionally, the cardiac risk factor (CRF) and the atherogenic index (AI) were calculated according to Kang et al. [22] as follows: (CRF = TC/HDL and AI = [TC − HDL]/HDL).

Immunological parameters included serum levels of interleukin (IL) 4 (IL-4), IL-10, IL-12, and interferon gamma (IFN-γ). All of the parameters were assessed calorimetrically (except for serum SAA, CRP, PCT, GSH, IL-4, IL-10, IL-12, and IFN-γ, which were assessed by sandwich enzyme-linked immunosorbent assay) according to the manufacturer’s instructions, using commercial kits from MyBioSource (San Diego, CA), except for those that assessed serum lipids, and liver and renal function, for which kits from SPINREACT (Girona, Spain) were used.

## Statistical analysis

Data were analyzed using the Statistical Package for Social Sciences version 22.0. (IBM, Armonk, NY). Quantitative data are presented as the mean and SD. The Shapiro–Wilk test was used to verify the normality of the distribution of variables. Since the data showed a normal distribution, parametric tests were used, with ANOVA (*F*-test) for the comparison of more than two means, followed by Tukey’s post hoc test for pairwise comparisons. The results are considered to be significant when *P* < 0.05, very significant when *P* < 0.01, and highly significant when *P* < 0.001.

## Results

### General assessment

At the end of the 8th week post-infection, the positive control group showed a statistically significant lower body weight (mean = 1.75 ± 0.37 kg) in comparison with the negative control group [mean = 4.68 ± 0.22 kg; ANOVA, *F*_(3, 36)_ = 81.314, *P* = 0.001]. Both IVM- and FLR-treated groups exhibited comparable body weight to that of the negative control group at the end of the 8th week post-treatment (mean = 4.53 ± 0.27 and 4.54 ± 0.31, respectively), with no statistically significant differences [ANOVA, *F*_(3, 36)_ = 98.06, *P* = 0.438 and 0.507, respectively].

### Clinical evaluation of cutaneous lesions

At the end of the 8th week post-infection, the infected non-treated rabbits exhibited marked lichenification, alopecia, hemorrhagic crusts, and fissures, mainly on limbs, ears, face, nose, and eyelids. Both treated groups showed complete clinical cure with resolution of all characteristic clinical lesions of sarcoptic mange at the end of the 8th week post-treatment.

### Parasitological findings

Microscopic examination of skin scrapings from the infected non-treated rabbits revealed the presence of large numbers of eggs, six-legged larvae, nymphs, and adult stages of *S. scabiei* (mean viable mite count = 501.4 ± 13.78). Mites were not detected in any skin scrapings at the end of the 8th week post-treatment in either treated group.

### Markers of systemic inflammation

The positive control group showed a highly significant increase in serum levels of LDH, CK-MB, SAA, and CRP in comparison with the negative control group [ANOVA, *F*_(3, 36)_ = 81.45, *P* = 0.001]. At the end of the 8th week post-treatment, the IVM-treated group showed a statistically significant rise in serum LDH [ANOVA, *F*_(3, 36)_ = 81.45, *P* = 0.037] and CK-MB [ANOVA, *F*_(3, 36)_ = 31, *P* = 0.005], a highly significant rise in serum CRP [ANOVA, *F*_(3, 36)_ = 189.68, *P* = 0.001], and a non-significant increase in SAA [ANOVA, *F*_(3, 36)_ = 1418.62, *P* = 0.171] in comparison with the negative control group. No statistically significant differences were recorded in the FLR-treated group regarding these parameters when compared with the negative control group [ANOVA, *F*_(3, 36)_ = 81.45, 31, 1418.62, and 189.68, *P* = 0.856, 0.965, 0.984, and 0.796 for LDH, CK-MB, SAA, and CRP, respectively] (Table 1).

**Table 1:** Mean values (± SD) of serum parameters of systemic inflammation

Parameter	Group	Mean ± SD	*F*-test	*P*-value	Post hoc test			
LDH (U/L)	Group I	169.84 ± 16.20	81.45	0.001***	*P*1	0.001***	*P*4	0.001***
	Group II	1311.06 ± 250.32			*P*2	0.856 NS	*P*5	0.001***
	Group III	363.28 ± 94.30			*P*3	0.037*	*P*6	0.05*
	Group IV	185.48 ± 23.54						
CK-MB (U/L)	Group I	302.40 ± 21.69	31.00	0.001***	*P*1	0.001***	*P*4	0.001***
	Group II	1238.76 ± 324.76			*P*2	0.965 NS	*P*5	0.001***
	Group III	661.54 ± 136.82			*P*3	0.005**	*P*6	0.006**
	Group IV	307.46 ± 25.93						
SAA (μg/ml)	Group I	3.55 ± 0.63	1418.62	0.001***	*P*1	0.001***	*P*4	0.001***
	Group II	116.39 ± 6.33			*P*2	0.984 NS	*P*5	0.001***
	Group III	6.56 ± 1.68			*P*3	0.171 NS	*P*6	0.177 NS
	Group IV	3.59 ± 0.91						
CRP (μg/ml)	Group I	5.95 ± 1.34	189.68	0.001***	*P*1	0.001***	*P*4	0.001***
	Group II	85.52 ± 7.57			*P*2	0.796 NS	*P*5	0.001***
	Group III	25.11 ± 9.34			*P*3	0.001***	*P*6	0.001***
	Group IV	6.96 ± 1.33						

*LDH* Lactate dehydrogenase, *CK-MB* creatine kinase myocardial band,* SAA* serum amyloid A, *CRP* C-reactive protein,* Group I* negative control,* Group II* positive control,* Group III* ivermectin (IVM)-treated group,* Group IV* fluralaner (FLR)-treated group, *P*1 group I and group II, *P*2 group I and group IV, *P*3 group I and group III, *P*4 group II and group IV, *P*5 group II and group III, *P*6 group IV and group III

*NS* (not significant) *P* > 0.05, * *P* < 0.05, ** *P* < 0.01, *** *P* < 0.001 (*n* = 10 for all groups)

### Serum PCT

The positive control group showed a highly significant increase in serum PCT in comparison with the negative control group [ANOVA, *F*_(3, 36)_ = 246.90, *P* = 0.001]. At the end of 8th week post-treatment, both IVM- and FLR-treated groups exhibited comparable results to those of the negative control group with no significant difference [ANOVA, *F*_(3, 36)_ = 189.68, *P* = 0.946 and 0.965, respectively] (Table 2).

**Table 2:** Mean values (± SD) of serum procalcitonin (*PCT*)

Parameter	Group	Mean ± SD	*F*-test	*P*-value	Post hoc test			
PCT (pg/ml)	Group I	14.80 ± 1.70	246.90	0.001***	*P*1	0.001***	*P*4	0.001***
	Group II	814.98 ± 113.60			*P*2	0.965 NS	*P*5	0.001***
	Group III	17.26 ± 3.24			*P*3	0.946 NS	*P*6	0.982 NS
	Group IV	16.42 ± 1.84						

For other abbreviations, see Table 1

*** *P* < 0.001 (*n* = 10 for all groups)

### Markers of redox status

The positive control group showed a highly significant increase in serum NO [ANOVA, *F*_(3, 36)_ = 62.414, *P* = 0.001] and MDA [ANOVA, *F*_(3, 36)_ = 33.858, *P* = 0.001], and a highly significant decrease in serum GSH [ANOVA, *F*_(3, 36)_ = 27.165, *P* = 0.001] and TAC [ANOVA, *F*_(3, 36)_ = 27.602, *P* = 0.001] in comparison with the negative control group. At the end of 8th week post-treatment, the IVM-treated group showed a highly significant rise in serum NO [ANOVA, *F*_(3, 36)_ = 62.414, *P* = 0.001], a statistically significant rise in serum MDA [ANOVA, *F*_(3, 36)_ = 33.858, *P* = 0.003], and a highly significant decrease in serum GSH [ANOVA, *F*_(3, 36)_ = 27.165, *P* = 0.001] and TAC [ANOVA, *F*_(3, 36)_ = 27.602, *P* = 0.001], when compared with the negative control group. No statistically significant differences were recorded in the FLR-treated group regarding these parameters when compared with the negative control group [ANOVA, *F*_(3, 36)_ = 62.414, 33.858, 27.165, and 27.602, *P* = 0.405, 0.907, 0.195 and 0.950 for NO, MDA, GSH, and TAC, respectively] (Table 3).

**Table 3:** Mean values (± SD) of serum redox status parameters

Parameter	Group	Mean ± SD	*F*-test	*P*-value	Post hoc test			
NO (μmol/L)	Group I	17.36 ± 4.69	62.414	0.001***	*P*1	0.001***	*P*4	0.001***
	Group II	78.93 ± 10.82			*P*2	0.405 NS	*P*5	0.001***
	Group III	49.03 ± 9.65			*P*3	0.001***	*P*6	0.001***
	Group IV	21.72 ± 5.26						
MDA (nmol/ml)	Group I	2.55 ± 0.14	33.858	0.001***	*P*1	0.001***	*P*4	0.001***
	Group II	11.46 ± 3.15			*P*2	0.907 NS	*P*5	0.001***
	Group III	6.15 ± 0.76			*P*3	0.003**	*P*6	0.003**
	Group IV	2.67 ± 0.25						
GSH (μg/ml)	Group I	7.11 ± 0.82	27.165	0.001***	*P*1	0.001***	*P*4	0.001***
	Group II	2.64 ± 0.64			*P*2	0.195 NS	*P*5	0.003**
	Group III	4.50 ± 0.75			*P*3	0.001***	*P*6	0.003**
	Group IV	6.37 ± 1.15						
TAC (mmol/l)	Group I	1.60 ± 0.25	27.602	0.001***	*P*1	0.001***	*P*4	0.001***
	Group II	0.47 ± 0.05			*P*2	0.950 NS	*P*5	0.047*
	Group III	0.80 ± 0.13			*P*3	0.001***	*P*6	0.001***
	Group IV	1.61 ± 0.40						

*NO* Nitric oxide,* MDA* malondialdehyde,* GSH* glutathione,* TAC* total antioxidant capacity; for other abbreviations, see Table 1

* *P* < 0.05, ** *P* < 0.01, *** *P* < 0.001 (*n* = 10 for all groups)

### Serum immunological parameters

The positive control group showed a highly significant increase in serum IL-4, IL-10, IL-12, and IFN-γ [ANOVA, *F*_(3, 36)_ = 552.21, 2606.33, 196.06, and 910.78, respectively, *P* = 0.001 for all]. At the end of 8th week post-treatment, the IVM-treated group showed a highly significant rise in serum IL-10, IL-12, and IFN-γ only [ANOVA, *F*_(3, 36)_ = 2606.33, 196.06, and 910.78, respectively, *P* = 0.001 for all], when compared with the negative control group. No statistically significant differences were recorded in the FLR-treated group regarding these parameters when compared with the negative control group [ANOVA, *F*_(3, 36)_ = 552.21, 2606.33, 196.06, and 910.78, *P* = 0.144, 0.141, 0.663, and 0.529 for IL-4, IL-10, IL-12, and IFN-γ, respectively] (Table 4).

**Table 4:** Mean values (± SD) of serum immunological parameters

Parameter	Group	Mean ± SD	*F*-test	*P*-value	Post hoc test			
IL-4 (pg/ml)	Group I	26.99 ± 3.10	552.21	0.001***	*P*1	0.001***	*P*4	0.001***
	Group II	206.83 ± 15.64			*P*2	0.144 NS	*P*5	0.001***
	Group III	35.08 ± 2.89			*P*3	0.143 NS	*P*6	0.997 NS
	Group IV	35.06 ± 3.67						
IL-10 (pg/ml)	Group I	170.84 ± 7.34	2606.33	0.001***	*P*1	0.001***	*P*4	0.001***
	Group II	816.87 ± 14.36			*P*2	0.141 NS	*P*5	0.001***
	Group III	301.54 ± 19.77			*P*3	0.001***	*P*6	0.001***
	Group IV	174.56 ± 7.52						
IL-12 (pg/ml)	Group I	209.28 ± 29.88	196.06	0.001***	*P*1	0.001***	*P*4	0.001***
	Group II	617.88 ± 20.23			*P*2	0.663 NS	*P*5	0.001***
	Group III	357.73 ± 32.25			*P*3	0.001***	*P*6	0.001***
	Group IV	200.55 ± 39.13						
IFN-γ (pg/ml)	Group I	26.07 ± 3.25	910.78	0.001***	*P*1	0.001***	*P*4	0.001***
	Group II	152.30 ± 5.15			*P*2	0.529 NS	*P*5	0.001***
	Group III	72.11 ± 4.64			*P*3	0.001***	*P*6	0.001***
	Group IV	27.85 ± 4.26						

*IL* Interleukin, *IFN-γ* interferon gamma; for other abbreviations, see Table 1

*** *P* < 0.001 (*n* = 10 for all groups)

### Assessment of the serum lipid profile

An atherogenic dyslipidemic profile was noted in the positive control group, as indicated by a highly significant increase in serum LDL, TG, and TC [ANOVA, *F*_(3, 36)_ = 444.293, 127.745, and 491.615, respectively, *P* = 0.001 for all], and a highly significant decrease in serum HDL [ANOVA, *F*_(3, 36)_ = 24.562, *P* = 0.001], compared with the normal group. A dyslipidemic profile was further supported by the highly significant increase in CRF and AI values in the positive control group [ANOVA, *F*_(3, 36)_ = 81.47 and 104.26, respectively, *P* = 0.001 for both], when compared with the negative control group. At day 56 post-treatment, no significant differences were noted regarding these parameters in either treated groups when compared with the negative control group (Tables 5, 6).

**Table 5:** Mean values (± SD) of the serum lipid profile

Parameter	Group	Mean ± SD	*F*-test	*P*-value	Post hoc test			
HDL (mg/dl)	Group I	27.19 ± 3.36	24.562	0.001***	*P*1	0.001***	*P*4	0.001***
	Group II	11.94 ± 1.67			*P*2	0.937 NS	*P*5	0.001***
	Group III	26.92 ± 3.97			*P*3	0.902 NS	*P*6	0.965 NS
	Group IV	27.01 ± 4.07						
LDL (mg/dl)	Group I	36.46 ± 4.65	444.293	0.001***	*P*1	0.001***	*P*4	0.001***
	Group II	176.33 ± 13.52			*P*2	0.842 NS	*P*5	0.001***
	Group III	33.79 ± 3.20			*P*3	0.579 NS	*P*6	0.453 NS
	Group IV	37.41 ± 2.73						
TG (mg/dl)	Group I	63.35 ± 9.08	127.735	0.001***	*P*1	0.001***	*P*4	0.001***
	Group II	230.68 ± 28.18			*P*2	0.798 NS	*P*5	0.001***
	Group III	72.35 ± 6.79			*P*3	0.393 NS	*P*6	0.545 NS
	Group IV	66.02 ± 11.18						
TC (mg/dl)	Group I	75.68 ± 5.15	491.615	0.001***	*P*1	0.001***	*P*4	0.001***
	Group II	234.04 ± 13.65			*P*2	0.839 NS	*P*5	0.001***
	Group III	75.97 ± 5.73			*P*3	0.954 NS	*P*6	0.885 NS
	Group IV	76.71 ± 2.81						

*HDL* High-density lipoprotein,* LDL* low-density lipoprotein,* TG* triglycerides,* TC* total cholesterol; for other abbreviations, see Table 1

*** *P* < 0.001 (*n* = 10 for all groups)

**Table 6:** Mean values (± SD) of the cardiac risk factor (*CRF*) and atherogenic index (*AI*)

Parameter	Group	Mean ± SD	*F*-test	*P*-value	Post hoc test			
CRF	Group I	2.80 ± 0.22	81.470	0.001***	*P*1	0.001***	*P*4	0.001***
	Group II	20.06 ± 4.24			*P*2	0.955 NS	*P*5	0.001***
	Group III	2.85 ± 0.25			*P*3	0.972 NS	*P*6	0.983 NS
	Group IV	2.88 ± 0.33						
AI	Group I	0.36 ± 0.08	104.260	0.001***	*P*1	0.001***	*P*4	0.001***
	Group II	1.28 ± 0.08			*P*2	0.743 NS	*P*5	0.001***
	Group III	0.43 ± 0.09			*P*3	0.306 NS	*P*6	0.480 NS
	Group IV	0.38 ± 0.12						

For other abbreviations, see Table 1

*** *P* < 0.001 (*n* = 10 for all groups)

### Assessment of hepatic structure and function

**Fig. 1 a–i:**
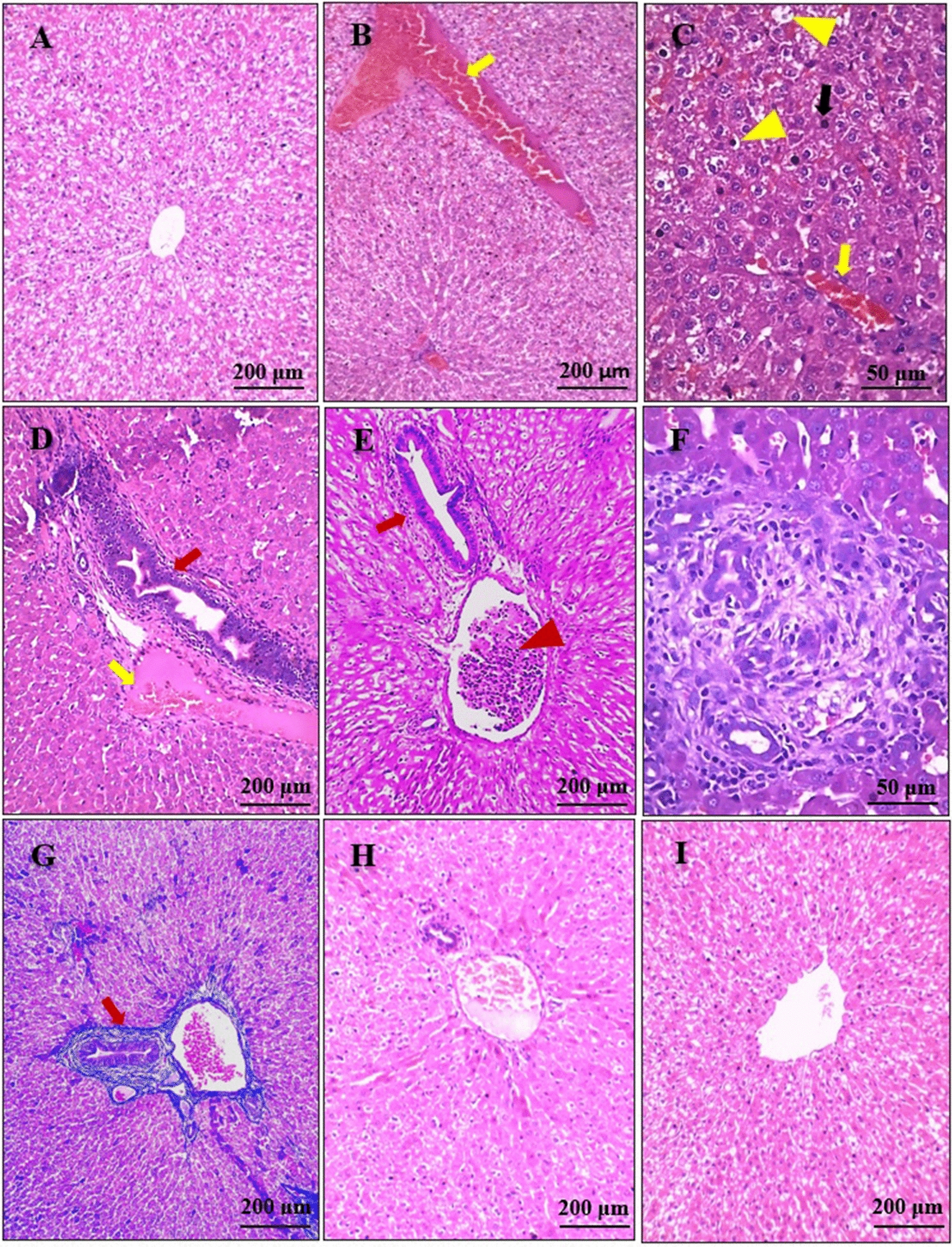
Photomicrographs of the liver sections. **a** Normal hepatic architecture. **b–g** Liver sections from the positive control group, showing marked dilatation and congestion (yellow arrow) of portal veins and hepatic sinusoids (**b**-**d**), various degrees of degenerative changes such as hydropic degeneration (yellow arrowhead) and pyknotic nuclei of hepatocytes (black arrow) (**c**), mild to moderate periportal inflammation and fibrosis (red arrows) (**d**–**g**), and pyemic thrombotic embolus within the portal vein (red arrowhead) (**e**). **h** Liver sections from the ivermectin (IVM)-treated rabbits, showing mild hepatic congestion and mild lymphocytic inflammation. **i** Nearly normal hepatic architecture in the FLR-treated group, with mild dilatation of the central vein. **a–f**, **h**, **i** H&E; **g** Masson’s trichrome

The liver sections from the positive control group showed marked congestion and dilatation of central veins, portal veins and hepatic sinusoids, pyemic thrombotic emboli within portal blood vessels, and various degrees of degenerative changes in hepatocytes, including mild hydropic degeneration and pyknotic hepatocytes. Moreover, mild to moderate periportal and perisinusoidal lymphocytic infiltrations were noted, accompanied with mild to moderate periportal fibrosis, evidenced by Masson's trichrome staining of liver sections. Hyperplasia of the epithelial lining of portal biliary radicals was also observed (Fig. 1b–g).

The liver sections of the IVM-treated group showed mild congestion and dilatation of central veins and blood sinusoids, as well as mild periportal lymphocytic inflammatory infiltrations, but at a much lower extent to that seen in the positive control group (Fig. 1h). The FLR-treated animals’ livers showed nearly normal hepatic histological architecture with only mild dilatation of the central veins (Fig. 1i).

The positive control group showed a highly significant decrease in serum albumin [ANOVA, *F*_(3, 36)_ = 23.709, *P* = 0.001] and a highly significant increase in serum ALT, AST, and ALP [ANOVA, *F*_(3, 36)_ = 97.226, 432.358, and 524.862, respectively, *P* = 0.001 for all]. At the end of the 8th week post-treatment, the IVM-treated group showed a statistically significant decrease in serum albumin [ANOVA, *F*_(3, 36)_ = 23.709, *P* = 0.006], a statistically significant increase in serum ALT [ANOVA, *F*_(3, 36)_ = 97.226, *P* = 0.014], and a highly significant increase in serum AST and ALP [ANOVA, *F*_(3, 36)_ = 432.358 and 524.862, respectively, *P* = 0.001 for both] when compared with the negative control group. No statistically significant differences were recorded for the FLR-treated group regarding these parameters when compared with the negative control group [ANOVA, *F*_(3, 36)_ = 23.709, 97.226, 432.358, and 524.862, *P* = 0.686, 0.581, 0.786, and 0.682 for albumin, ALT, AST, and ALP, respectively] (Table 7).

**Table 7:** Mean values (± SD) of parameters of liver and renal function tests

Parameter	Group	Mean ± SD	*F*-test	*P*-value	Post hoc test			
Albumin (g/dl)	Group I	4.41 ± 0.56	23.709	0.001***	*P*1	0.001***	*P*4	0.001***
	Group II	2.23 ± 0.18			*P*2	0.686 NS	*P*5	0.001***
	Group III	3.48 ± 0.49			*P*3	0.006**	*P*6	0.013*
	Group IV	4.29 ± 0.50						
ALT (U/L)	Group I	57.20 ± 7.62	97.226	0.001***	*P*1	0.001***	*P*4	0.001***
	Group II	188.51 ± 24.40			*P*2	0.581 NS	*P*5	0.001***
	Group III	81.56 ± 8.04			*P*3	0.014*	*P*6	0.044*
	Group IV	62.18 ± 8.01						
AST (U/L)	Group I	26.63 ± 3.75	432.358	0.001***	*P*1	0.001***	*P*4	0.001***
	Group II	163.12 ± 12.03			*P*2	0.786 NS	*P*5	0.001***
	Group III	44.56 ± 4.88			*P*3	0.001***	*P*6	0.002**
	Group IV	27.87 ± 4.03						
ALP (U/L)	Group I	56.91 ± 3.40	524.862	0.001***	*P*1	0.001***	*P*4	0.001***
	Group II	189.60 ± 10.47			*P*2	0.682 NS	*P*5	0.001***
	Group III	79.00 ± 4.03			*P*3	0.001***	*P*6	0.001***
	Group IV	56.22 ± 4.13						
BUN (mg/dl)	Group I	24.91 ± 2.42	47.343	0.001***	*P*1	0.001***	*P*4	0.001***
	Group II	37.67 ± 3.32			*P*2	0.174 NS	*P*5	0.001***
	Group III	17.57 ± 2.15			*P*3	0.001***	*P*6	0.015*
	Group IV	22.40 ± 3.09						
Cr (mg/dl)	Group I	0.74 ± 0.08	184.874	0.001***	*P*1	0.001***	*P*4	0.001***
	Group II	1.94 ± 0.04			*P*2	0.052 NS	*P*5	0.001***
	Group III	1.15 ± 0.08			*P*3	0.001***	*P*6	0.001***
	Group IV	0.86 ± 0.13						

*ALT* Alanine aminotransferase,* AST* aspartate aminotransferase,* ALP* alkaline phosphatase,* BUN* blood urea nitrogen,* Cr* creatinine; for other abbreviations, see Table 1

* *P* < 0.05, ** *P* < 0.01, *** *P* < 0.001 (*n* = 10 for all groups)

### Assessment of kidney structure and function

**Fig. 2 a–i:**
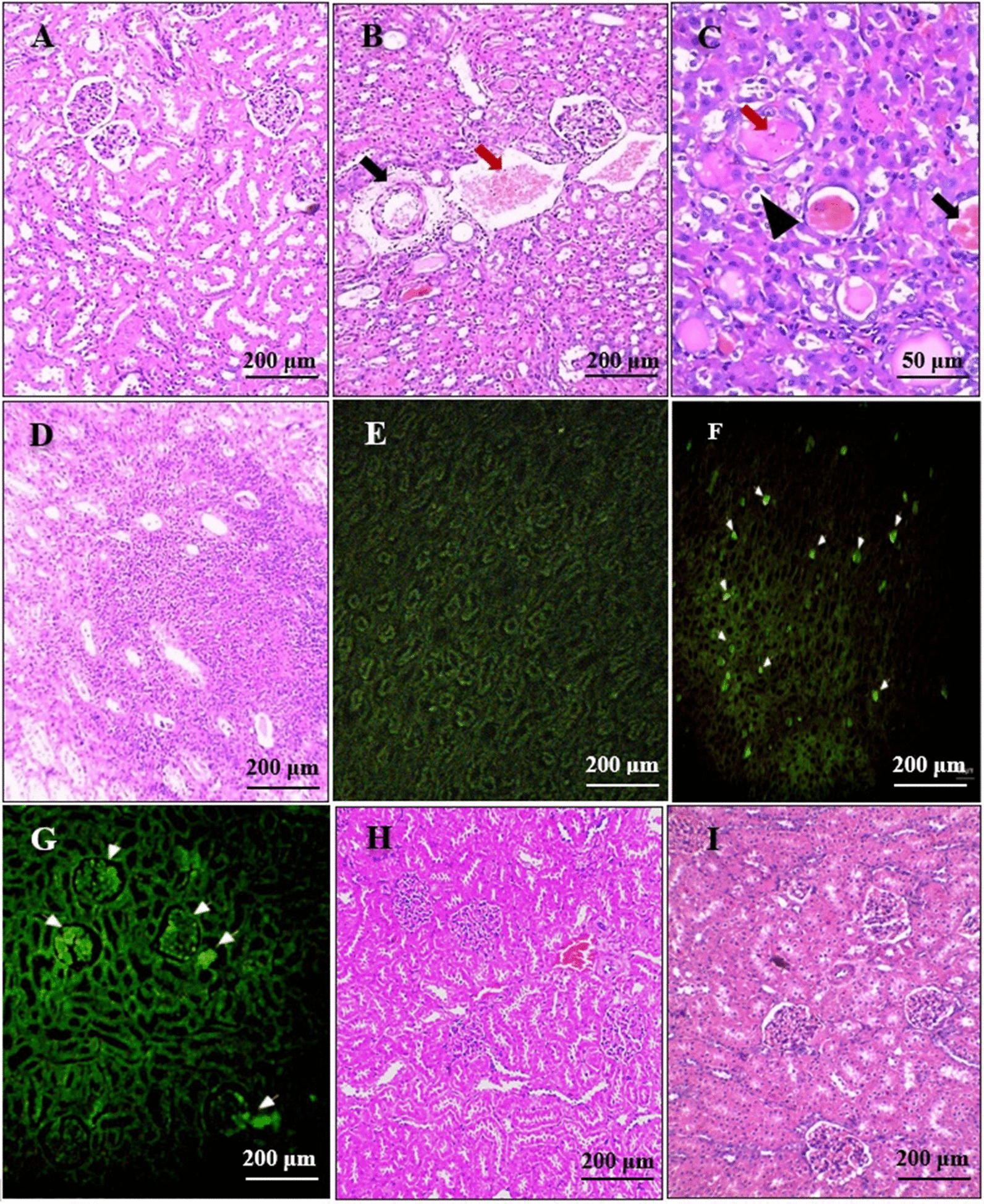
Photomicrographs of the renal sections. **a** Normal renal histological architecture. **b**–**g** Sections from the positive control group that show severe renal congestion (red arrow), thick-walled vessels (black arrow) (**b**), tubular casts (red arrows), vacuolar and hydropic degeneration of tubular cells (black arrowhead), tubular hemorrhage (black arrow) (**c**), moderate to severe peritubular inflammation (**d**), and bright greenish-yellow fluorescence at the sites of immune complex deposition (**f**, **g**). **e** Negative control slide that was not treated with serum during the immunofluorescence staining procedure. **h** A renal section from the IVM-treated group, showing mild congestion with a few inflammatory peritubular infiltrates. **i** Renal sections from the FLR-treated group, showing normal renal histological architecture. **a**–**d**, **h**, **i** H&E; **e**–**g** immunofluorescence anti-immunoglobulin G. For abbreviations, see Fig. 1

The renal sections from the positive control group revealed moderate to severe congestion, thickening of the vascular walls, perivascular fibrosis (some blood vessels were even obliterated by fibrosis), degenerative changes of tubular cells (mainly vacuolar and hydropic degeneration), and moderate to severe peritubular inflammatory infiltration (mainly by neutrophils, lymphocytes, and plasma cells). Eosinophilic hyaline casts were also observed within tubules (Fig. 2b–d). Furthermore, bright greenish-yellow fluorescence was noted in the immunofluorescent-stained renal sections of the positive control group at the sites of immune complex deposition (Fig. 2e–g).

Renal sections from the IVM-treated group showed fewer peritubular lymphocytic inflammatory infiltrations with mild congestion of the renal blood vessels (Fig. 2h). Regarding rabbits treated with FLR, the kidney sections showed nearly normal histological architecture (Fig. 2i).

The positive control group showed a highly significant increase in serum BUN and Cr [ANOVA, *F*_(3, 36)_ = 47.343 and 184.874, respectively, *P* = 0.001 for both]. At the end of the 8th week post-treatment, the IVM-treated group still showed a highly significant increase in both of these parameters in comparison with the normal group [ANOVA, *F*_(3, 36)_ = 47.343 and 184.874, respectively, *P* = 0.001 for both], while the levels in the FLR-treated group were comparable to those in the normal group [ANOVA, *F*_(3, 36)_ = 47.343 and 184.874, *P* = 0.174 and 0.052, respectively] (Table 7).

### Other histopathological findings

**Fig. 3 a–e:**
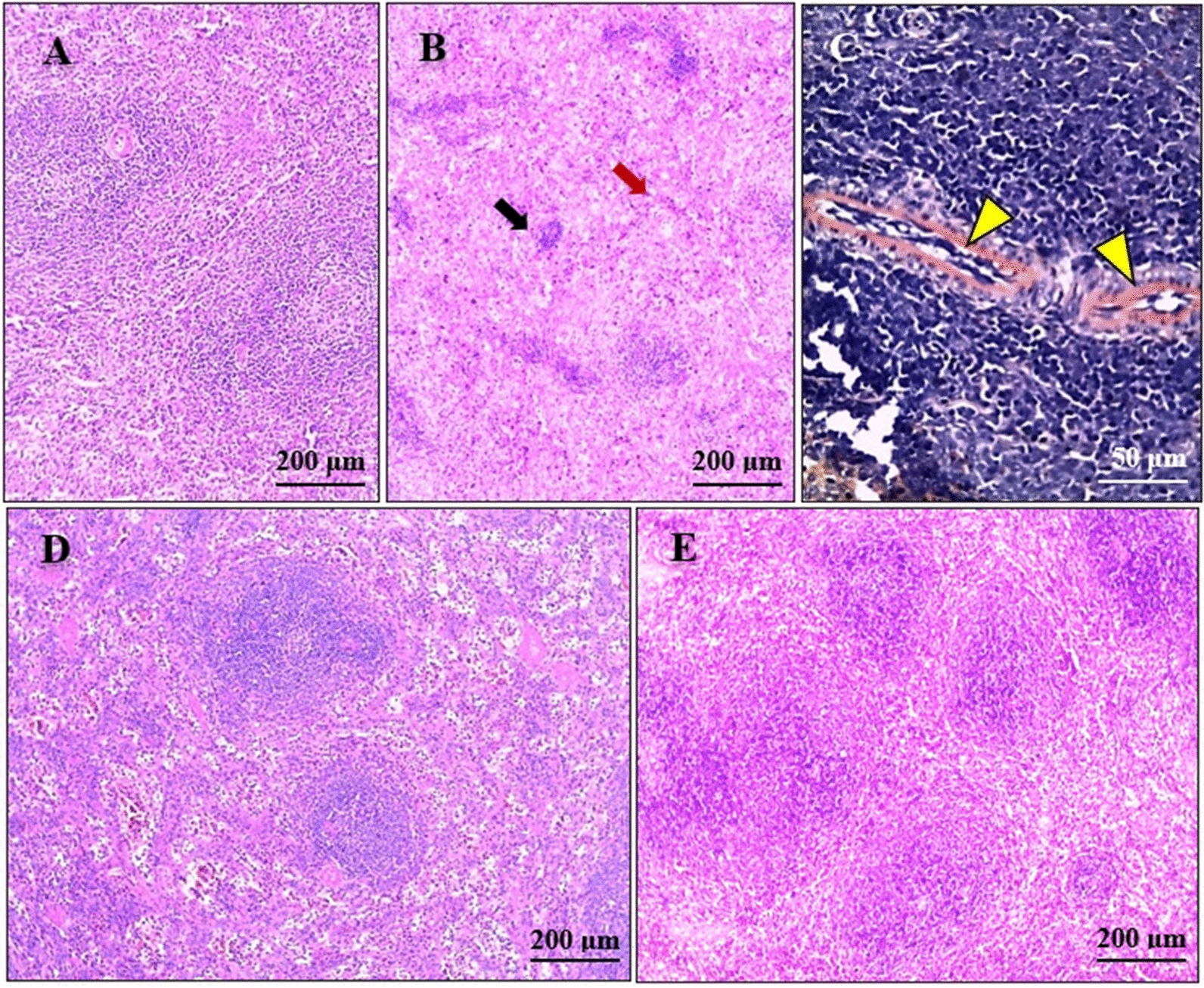
Photomicrographs of the spleen sections. **a** Normal spleen histological architecture. **b**, **c** Sections from the positive control group, showing moderate to severe splenic congestion in the red pulp (red arrow), white pulp atrophy (black arrow) (**b**), peri-vascular amyloid deposits (yellow arrowheads) (**c**). **d** Splenic sections from the IVM-treated group, showing moderate congestion and partial improvement of the white pulp. **e** Splenic sections from the FLR-treated group, showing nearly normal splenic histological architecture with mild congestion of the red pulp. **a**, **b**, **d**, **e** H&E, **c** Congo red

The splenic sections from the positive control group exhibited moderate to severe congestion of the red pulp, marked atrophy of the white pulp, and perivascular amyloid deposits, evidenced by amorphous reddish discoloration on Congo red staining (Fig. 3b, c). The splenic sections of the IVM-treated rabbits revealed just mild to moderate congestion with a notable improvement of the white pulp (Fig. 3d). Sections from the FLR-treated group showed nearly normal architecture (Fig. 3e).

**Fig. 4 a–e:**
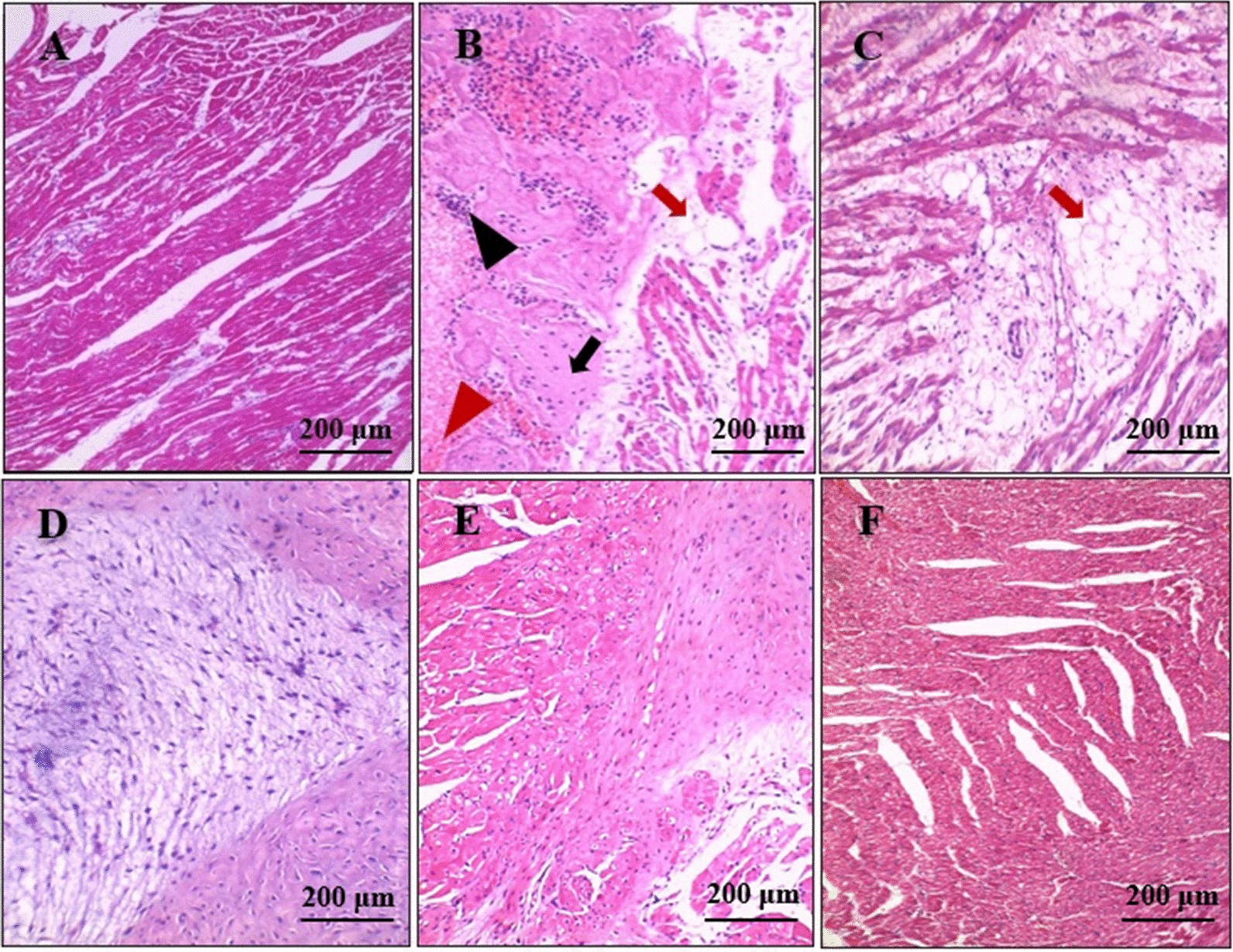
Photomicrographs of the heart sections.** a** Normal heart histological architecture.** b–d** Sections from the positive control group, showing moderate congestion (red arrowhead), inflammatory neutrophilic infiltration (black arrowhead), hyaline degeneration (black arrow) (**b**), fatty infiltration of the cardiac muscles (red arrow) (**b,** **c**), and myxomatous degeneration of the cardiac muscles (**d**). **e** Cardiac sections from the IVM-treated group, showing occasional mild myxomatous degeneration. **f** Cardiac sections from the FLR-treated group, showing nearly normal cardiac histological architecture (H&E). For abbreviations, see Fig. 1

The cardiac sections from the positive control group showed moderate to severe congestion, myocardial degenerative changes (mainly hyaline and myxomatous degeneration), fatty infiltration of the cardiac muscles, and mild to moderate inflammatory cell infiltration (mainly neutrophils, lymphocytes, and eosinophils) (Fig. 4b–d). Sections from the IVM-treated group showed mild congestion and occasional myxomatous degeneration of the cardiac muscles (Fig. 4e). Meanwhile, sections from the FLR-treated group showed no significant pathological changes (Fig. 4f).

## Discussion

In this study, non-treated rabbits with experimentally induced CS were used as a positive control group to study systemic changes induced by CS, while both IVM- and FLR-treated groups were used to reveal the impact of treatment on these changes. Several parameters were used in our study in an attempt to understand the disease’s evolution and to unravel the underlying factors that could explain how CS might affect the host's general condition or even endanger its life.

Because experimental studies of CS in humans are difficult and ethically problematic, our study was performed using *S. scabies* var. *cuniculi* instead of *S. scabies* var. *hominis*, and rabbits were used as the experimental model since they are suitable for the collection of a large number of mites and they are highly acceptable models for research purposes. Additionally, they develop clinical responses to *S. scabiei* infestation that are similar to those seen in humans, and a form of the disease that is very similar to that seen in humans [23].

Weight loss noted in the positive control group in our study could be attributed to the inflammatory state induced by the widespread mite infestation, which can alter metabolism and food utilization, leading to increased catabolism of muscle proteins [24]. Itching, nausea induced by ear infection, and pain on chewing caused by the thick incapacitating crusts around the mouth are possible additional factors that could explain why mangy animals exhibit low food intake and emaciation. Interestingly, scabies-induced malnutrition and loss of macro- and/or micronutrients could partly explain the structural and functional changes recorded in our study.

Although a Th2 immune response has been reported as the main immune response in CS [25], our study revealed a mixed cytokine profile, evidenced by increased serum levels of IL-4, IL-10, IL-12, and IFN-γ in the positive control group. These findings are in accordance with De et al. [7], who reported a significant increase in both proinflammatory cytokines (IL-1β, IL-2, IL-6, IL-12, and IFN-γ) and anti-inflammatory cytokine IL-4 in mangy pigs, indicating a mixed Th1/Th2 immune response. De et al. [7] explained that such a disorganized immune response was due to the modulation of immunity by infection with *S. scabiei* or changes in cytokine levels when the infection shifted to the chronic phase. Hence, differences in the timing of the collection of blood samples in relation to disease progression could partly explain disparities in the results of immune assays between different studies. Additionally, the presence or absence of secondary infections, oxidative stress-induced inflammation, disease severity, nature of the host, and host immune status are all important factors.

Interestingly, the saliva of the mite contains bioactive substances that can affect the host's immune system in various ways. Although they cannot function as antigens, low molecular weight salivary components can attach to skin proteins as haptens and trigger a Th1 response. Meanwhile, salivary antigens may potentially induce a Th2 response together with the synthesis of IgE and type I hypersensitivity [26]. Mounsey et al. [27] reported that Th2 responses in CS dominate the immune response prior to the development of high mite burdens and before major clinical or histological changes became evident. Generally, the immune response against *S. scabiei* is extremely complicated, and meticulous sequential analysis of the local and systemic immune responses is recommended.

Although no microbiological analyses were carried out in the current work, an increase in serum PCT can be considered indirect evidence of secondary bacterial infection, which could be a major contributing factor for all the pathological changes recorded in our study. Bacterial lipopolysaccharides are widely known to be the most effective inducers of PCT secretion. In contrast to conventional inflammatory markers like CRP, serum PCT has higher sensitivity and specificity in bacterial infections and does not increase in patients with sterile inflammation or viral infection [28]. The current work revealed that there was a significant increase in serum PCT in the positive control group. This finding was consistent with the fact that infection with *S. scabiei* is usually associated with pruritus and traumatic skin injuries, which in turn can increase the likelihood of secondary bacterial infections [29].

A wide variety of bacterial agents were isolated from mangy ibex by Espinosa et al. [30]. The authors reported a septicaemic pattern of infection in their study, and the bacterial agents (mainly *Staphylococcus*) were isolated from the mangy skin and suppurative lesions in lymph nodes and various organs. Although it may be difficult to distinguish the contribution of the mites from those of the accompanying bacterial agents to the pathogenesis of CS, well-designed controlled studies may allow researchers to better elucidate the pathogenesis of the disease. Furthermore, the administration of antibiotics in addition to a scabicidal agent seems reasonable for the treatment of severe cases of CS.

Although levels of acute-phase proteins (APPs) can be expected to be elevated in the early phases of infection (i.e., mild and moderate cases) and slowly wane with chronic infection (i.e., severe cases of CS) [31], serum levels of SAA and CRP were increased in the positive control group in our study. These findings are in accordance with those of Dinler et al. [32] in their study of mangy dogs. Proinflammatory cytokines can initiate an acute phase reaction in the host, causing increased levels of APPs in the serum. This finding coincides with the recorded increases in serum IL-12 and INF-γ in the positive control group in our study. The magnitude of increase in serum APPs is generally related to the extent of infection and tissue damage. Interestingly, Dinler et al. [32] reported that serum SAA was a better sign reflecting the severity of sarcoptic mange in dogs than other APPs, whereas CRP was superior to other APPs for the detection of cases complicated by superficial pyoderma.

The state of oxidative stress in the positive control group in our study, as evidenced by increased serum NO and MDA and decreased serum GSH and TAC, was in accordance with a study performed on mangy pigs [7]. Mange-induced release of proinflammatory cytokines and inflammatory reactions could explain such findings. Under oxidative stress, several antioxidant enzymes, including GSH, are consumed by the body to fight against free radicals and maintain homeostasis [33]. Interestingly, Khalafalla et al. [34] reported increased blood TAC in mangy rabbits and attributed this to the initial activation of the antioxidative processes that usually precede the exhaustion of such homeostatic defenses.

Such changes in serum markers of redox status in our study indicated that oxidative stress in mange may be a systematic syndrome that could affect other tissues besides those of the skin. At high levels, reactive oxygen species and reactive nitrogen species can trigger damage to lipids, proteins, lipoproteins, and nucleic acids, leading to possible structural and functional changes in various organs [35]. Importantly, such a syndrome is more likely to occur in severe cases of CS. Various biochemical and histopathological changes recorded in the positive control group in our study were consistent with this assumption.

Highly significant increases in serum levels of LDH and CK-MB were noted in the positive control group. De et al. [7] reported similar results in mangy pigs and attributed this to mange-induced oxidative stress. That finding was consistent with the histopathological findings for the hepatic and cardiac sections from the positive control group in our study. LDH is a tetrameric enzyme found mainly in muscles, liver, and erythrocytes. Its increase in serum could be a sign of cellular death and enzyme leakage from cells [36]. Importantly, the CK-MB enzyme is found primarily in heart muscle cells and to a lesser extent in the skeletal muscles. Hence, a significant increase of serum CK-MB is more likely to be related to cardiac muscle injury than to skeletal muscle injury [37].

The positive control group exhibited definite hepatic and renal histopathological changes, with deviant values of serum albumin, ALT, AST, ALP, BUN, and Cr. It is not clear which factors specifically caused such changes, although mite products (such as salivary toxins and antigens), circulating immune complexes, oxidative stress, and even secondary bacterial infections are all possible ones. This is consistent with the oxidative stress condition, increased serum PCT, and immune complex deposition seen in the renal tissues in the positive control group in our study.

Interestingly, Villalba-Briones et al. [38] reported that, under microscopic analysis, liver and renal sections from mangy wild coati showed slight centrilobular congestion, with no other obvious pathological changes. In another study, on mangy Iberian lynx, Oleaga et al. [39] reported renal inflammatory and degenerative changes. De et al. [7] reported increased ALT, AST, ALP in mangy pigs, with decreased serum albumin, while Nwufoh et al. [29] reported that there was no significant increase in serum ALT and AST in mangy dogs included in their study. Moreover, Upadhyay et al. [40] reported a significant decrease in serum BUN only in mangy dogs. Differences in the severity and duration of the disease could explain variations in results reported in different studies. It appears that only severely affected animals exhibit such changes. The nature of the host, presence or absence of co-infections, and host immune status are, once again, important additional factors.

Generally, increased serum ALT, AST, and ALP in the positive control group in our study were consistent with the histopathological changes in the liver sections from the same group, and, as McGill [41] reported, significant elevations in these enzymes may indicate liver inflammation, hepatocyte damage, and possibly biliary stasis. Similarly, in our study, the observed significant increases in serum BUN and serum Cr in the positive control group were consistent with the histopathological changes that were recorded in the same group. Increased serum BUN may be attributed to protein catabolism secondary to starvation or infection. Additionally, defective renal perfusion could influence the excretory functions of the kidney, leading to increased values of serum BUN.

Along similar lines, we can assume that immune-mediated glomerulonephritis secondary to the superimposed bacterial infections, or possibly mite-related immune complex deposition, could be another factor that explains an increase in serum BUN. The decreased serum BUN in some reports could be attributed to loss of appetite, hepatic insufficiency (that may have led to decreased protein catabolism), and/or reduced protein deposition, particularly by wasting muscle in emaciated animals. It appears that changes in renal function are multifactorial and depend on the severity of infestation, extent of hepatic and renal pathology, and nutritional status of the affected animals.

The reported decrease in serum albumin in the positive control group could be explained by the loss of plasma proteins from the exudative dermatitis and the persistent sucking of fluid by the mites, impaired liver function, and oxidative stress. Interestingly, albumin, which is known to be the major extracellular source of thiols, can limit the production of reactive oxygen species by binding free copper (which is especially important in accelerating the production of free radicals) [42].

The histopathological changes reported in the spleen sections taken from the infected non-treated rabbits were generally similar to those reported for Iberian ibex by Espinosa et al. [30]; however, those authors reported that there was hyperplasia of splenic lymphoid follicles with formation of germinal centers. In contrast, Villalba-Briones et al. [38] reported that no obvious pathological changes were found in the spleen of mangy wild coati in their study. The amyloid deposits noted in the splenic sections from the positive control group in our study are consistent with the significantly increased serum SAA in the same group, and as Hooijberg et al. [31] reported, the chronic expression of SAA can result in amyloid deposits in different tissues.

The atherogenic dyslipidemic profile noted in the positive control group was in accordance with that seen in a study by De et al. [7] of mangy Nicobari pigs. These authors suggested that CS could lead to a shift in the lipid profile towards atherogenic dyslipidemia, resulting in a high susceptibility of the affected animals to cardiovascular disorders. AI is considered a better indicator of coronary heart disease risk than individual lipoprotein concentrations [43]. Chronic inflammation in animals with CS could explain their aberrant lipid profiles. Moreover, oxidative stress might be the underlying cause of dyslipidemia, since increased serum levels of proinflammatory cytokines such as IL-6 and IFN-γ in infested animals could inhibit the activity of lipoprotein lipase which hinders the clearance of very LDL and LDL cholesterol leading to their increased concentration in blood [44]. Further studies are needed to investigate the role of CS as a possible risk factor for atherosclerosis and cerebrovascular accidents in humans.

Although the IVM-treated group showed clinical and parasitological cure, our study revealed persistent mild histopathological changes in the internal organ tissue sections that we examined, with persistent mild increases in serum LDH, CK-MB, CRP, NO, MDA, ALT, AST, ALP, BUN, Cr, IL-10, IL-12, and IFN-γ, together with persistent mild decreases in serum GSH, TAC, and albumin, when compared with the normal group. Since both clinical and parasitological cure were noted together with the normalization of serum PCT, we could assume that these changes were induced by the drug itself (not by the mite or secondary bacterial infections).

It seemed that IVM-induced oxidative stress caused tissue injury, with subsequent enzyme leakage from the injured cells, an inflammatory response, and acute phase reaction. These responses are consistent with the results of Abdel-Rahman and Ali [45], who reported that IVM-treated ewes showed a significant decrease in the activity of GSH and TAC for up to 3 months post-treatment. Abu Hafsa et al. [24] reported that the state of oxidative stress and the toxic effects of IVM on different tissues such as the liver and kidney were quite long-lasting and required at least 3 months to resolve, but could be minimized or even disappear upon the use of antioxidants like turmeric extract concurrently with IVM therapy. Generally, IVM is described as a safe drug if used at the recommended dose, but toxic effects may become apparent if doses are repeated or if the dose is increased [46].

Interestingly, the recorded improvement in the lipid profile in the IVM-treated group in the current study was in accordance with Chahrazed et al. [47], who reported that IVM therapy induced a significant decrease in all lipid parameters, with the exception of HDL (which was increased). Jin et al. [48] reported that IVM, a farnesoid X receptor ligand that regulates gene expression to maintain bile acid and cholesterol homeostasis, can successfully alleviate hyperlipidemia in diabetic mice models.

Our study revealed that a single dose of FLR was able to induce clinical and parasitological cure in rabbits with CS without the need for repeated doses. According to pharmacokinetic studies, FLR has a much longer duration of action after a single dose has been administered compared to IVM. This is particularly advantageous in treating scabies, in which the use of drugs that require multiple dosing could be challenging [49]. Interestingly, the FLR-treated group exhibited comparable results of all serum biochemical and immunological parameters to those of the normal control, with nearly normal histological sections for all examined internal organs, which suggested higher efficacy and safety of FLR over IVM in the treatment of the systemic effects of sarcoptic mange.

According to the European Medicines Agency [50], FLR is safe for use in rats when given as a single dose; however, some histopathological changes were noted in the lung, thymus, and liver when the drug was given to rats at a repeat dose of 400–600 mg/kg. It is not yet clear whether these findings were simply due to the elimination of the detrimental effects of the mite infection or additional, unknown actions of the drug beside its scabicidal effect. In a safety study performed on bare-nosed wombats, Wilkinson et al. [13] reported that there was a significant decrease in TC in FLR-treated animals. Further studies are needed to investigate the possible effects of FLR on the serum lipid profile of FLR-treated animals.

To the best of our knowledge, this is the first study to assess the efficacy of FLR in the treatment of CS at the systemic level, and to report a complete reversal of all recorded structural and functional changes in infected treated rabbits. Additionally, it shows that such changes can be reversed when an appropriate scabicide is used.

One of the limitations of our study was the poor general condition of the studied rabbits in the positive control group, which resulted in the relatively early timing of assessment (8 weeks post-infection) to avoid their sudden death. Additionally, systemic changes in rabbits may not be fully representative of those that may occur in larger animal models. Hence, it is recommended that well-controlled studies should be undertaken to examine the situation in bigger animal models like pigs, and in humans. Our study showed that the impact of CS on the host involves complex interconnected factors, and that it is not easy to distinguish them. Hence, more well-designed and controlled studies are needed to address this.

## Conclusions

Our study showed that CS in the rabbit model is a systemic syndrome that can affect other organs besides the skin. This syndrome is more likely to occur in severe cases of scabies. It appears that mite antigens and secondary bacterial agents could induce different immune responses and inflammatory reactions, with a subsequent alteration of the redox status of the affected host. The resulting oxidative stress condition could induce damage in different tissues, with subsequent changes in serum biochemical and immunological parameters. Since most of these parameters reverted to normality or almost normal levels after elimination of the mites, it can be concluded that the changes were induced by the infestation and not by other health-related factors. Additionally, it appears that FLR is a better choice for the alleviation of the local and systemic consequences of CS. Hence, it appears that FLR is a promising scabicidal agent for possible use in humans, and we recommend that this should be examined in future studies.

### Supplementary Information

**Additional file 1.** Systemic changes in crusted scabies.

## Data Availability

The authors confirm that the data supporting the findings in this study are available within the article and its supplementary material. Raw data that support the findings of this study are available from the corresponding author, upon reasonable request.

## Abbreviations

AI: Atherogenic index
ALP: Alkaline phosphatase
ALT: Alanine aminotransferase
APP: Acute-phase protein
AST: Aspartate aminotransferase
BUN: Blood urea nitrogen
CK-MB: Creatine kinase myocardial band
Cr: Creatinine
CRF: Cardiac risk factor
CRP: C-reactive protein
CS: Crusted scabies
FLR: Fluralaner
GSH: Glutathione
HDL: High-density lipoprotein
IFN-γ: Interferon gamma
IL: Interleukin
IVM: Ivermectin
LDH: Lactate dehydrogenase
LDL: Low-density lipoprotein
MDA: Malondialdehyde
NO: Nitric oxide
PCT: Procalcitonin
SAA: Serum amyloid A
TAC: Total antioxidant capacity
TC: Total cholesterol
TG: Triglycerides
Th: Helper T cell
