# Supplementary material for: Crusted scabies in a rabbit model: a severe skin disease or more?
Source: Parasit Vectors. 2023 Nov 14;16:413. doi: 10.1186/s13071-023-05995-8 (PMC10647032; doi:10.1186/s13071-023-05995-8)

Crusted scabies: insights beyond the skin

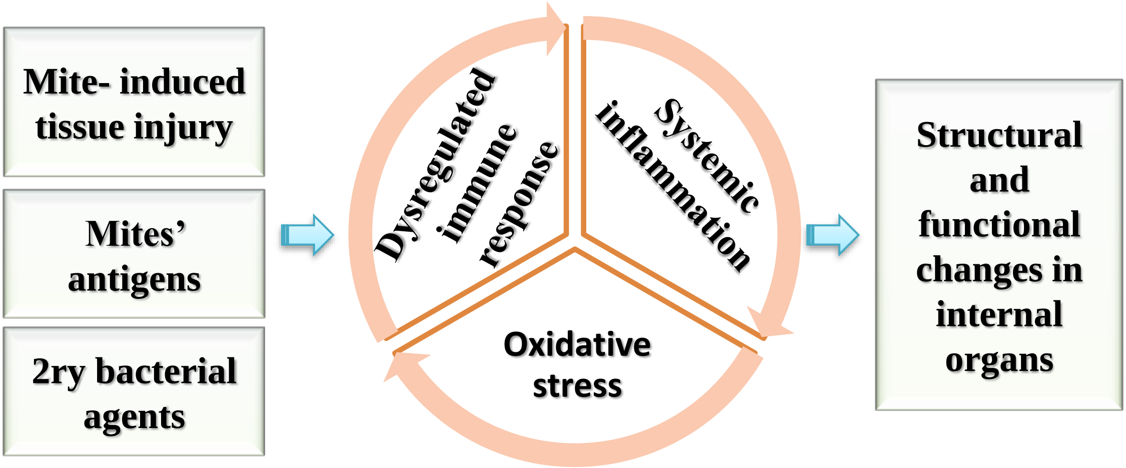

Supplement: Supplementary file 1 — Additional file 1. Systemic changes in crusted scabies. [file 13071_2023_5995_MOESM1_ESM.pdf]
